# Identification of Poly(ADP-Ribose) Polymerase-1 as a Cell Cycle Regulator through Modulating Sp1 Mediated Transcription in Human Hepatoma Cells

**DOI:** 10.1371/journal.pone.0082872

**Published:** 2013-12-19

**Authors:** Liu Yang, Kun Huang, Xiangrao Li, Meng Du, Xiang Kang, Xi Luo, Lu Gao, Cheng Wang, Yanqing Zhang, Chun Zhang, Qiangsong Tong, Kai Huang, Fengxiao Zhang, Dan Huang

**Affiliations:** 1 Department of Cardiovascular Diseases, Union Hospital, Tongji Medical College, Huazhong University of Science and Technology, Wuhan, China; 2 Clinical Center for Human Genomic Research, Union Hospital, Huazhong University of Science and Technology, Wuhan, China; 3 Department of Obstetrics and Gynecology, Union Hospital, Tongji Medical College, Huazhong University of Science and Technology, Wuhan, China; Johns Hopkins University, United States of America

## Abstract

The transcription factor Sp1 is implicated in the activation of G0/G1 phase genes. Modulation of Sp1 transcription activities may affect G1-S checkpoint, resulting in changes in cell proliferation. In this study, our results demonstrated that activated poly(ADP-ribose) polymerase 1 (PARP-1) promoted cell proliferation by inhibiting Sp1 signaling pathway. Cell proliferation and cell cycle assays demonstrated that PARP inhibitors or PARP-1 siRNA treatment significantly inhibited proliferation of hepatoma cells and induced G0/G1 cell cycle arrest in hepatoma cells, while overexpression of PARP-1 or PARP-1 activator treatment promoted cell cycle progression. Simultaneously, inhibition of PARP-1 enhanced the expression of Sp1-mediated checkpoint proteins, such as p21 and p27. In this study, we also showed that Sp1 was poly(ADP-ribosyl)ated by PARP-1 in hepatoma cells. Poly(ADP-ribosyl)ation suppressed Sp1 mediated transcription through preventing Sp1 binding to the Sp1 response element present in the promoters of target genes. Taken together, these data indicated that PARP-1 inhibition attenuated the poly(ADP-ribosyl)ation of Sp1 and significantly increased the expression of Sp1 target genes, resulting in G0/G1 cell cycle arrest and the decreased proliferative ability of the hepatoma cells.

## Introduction

Specificity protein 1 (Sp1) was the first transcription factor identified and cloned in mammalian [1]. It belongs to the Sp/XKLF (Specificity protein/Krüppel-like factor) family, which has been implicated in a host of essential biological processes. The Sp1 protein comprises several domains, including N-terminal inhibitory domain, serine/threonine-rich domains, glutamine-rich domains, zinc finger DNA binding domain, and the C-terminal DNA binding domain. The Ser/Thr-rich region is crucial in the regulation of Sp1 and could be regulated by post-modification. The C-terminal DNA binding domain of Sp1 consists of three contiguous Zn fingers binding motifs required for recognizing GC boxes located in the target gene promoters [2], [3]. Previous studies have indicated that regulation of Sp1-dependent transcription can be dramatically affected by changes in its DNA binding activity or transcriptional activity [4]. It has also been proposed that Sp1 is essential for the transcription of various genes, such as INK4 (including p15, p16, p18 and p19) and Cip/Kip (such as p21 and p27) family genes, which induce cell cycle arrest at G0/G1 phase [5], [6], [7], [8], [9], [10]. Thus, Sp1 plays a critical role in diverse processes, including cell cycle, cell proliferation and apoptosis [11], [12], [13], [14], [15], [16], [17].

Poly(ADP-ribose) polymerase-1 (PARP-1) is an ubiquitous nuclear DNA base repair enzyme present in eukaryotes. As the most abundant member of PARP family, PARP-1 accounts for about 90% of total cellular PARP activity. In nucleus, activated PARP-1 catalyses the transfer of ADP-ribose from nicotinamide adenine dinucleotide (NAD^+^) onto nuclear acceptor proteins [18], [19]. This process known as poly(ADP-ribosyl)ation causes chromatin relaxation and functions as a scaffold that facilitates the recruitment and assembly of the DNA repair proteins [20], [21]. As polymer chains can reach more than 200 units on the acceptor, poly(ADP-ribosyl)ation may result in remarkable conformational change of the acceptor protein [19], thereby functioning importantly in diverse biological processes, including transcriptional regulation, chromatin remodeling, DNA repair, cell proliferation, and apoptosis [18]. Several studies have shown that PARP-1 acts as a mediator of cell cycle due to its function as a regulator of various transcriptional factors, such as E2F-1, FOXO1 and c-Fos [22], [23], [24], [25]. We then explored the role of PARP-1 in the Sp1-mediated cell cycle arrest.

In the present study, in order to clarify the impact of PARP-1 in cell growth and cell cycle progression, we investigated the effects of pharmacologic PARP-1 inhibitors 3-aminobenzamide (3AB) and N-(6-oxo-5, 6-dihydrophenanthridin-2-yl)-2-(N, N-dimethylamino)acetami (PJ34), enzymatic PARP-1 activator H_2_O_2_, PARP-1 siRNA, as well as PARP-1 expressing plasmid on proliferation and cell cycle distribution of human hepatoma cells. Inhibition of PARP-1 significantly suppressed human hepatoma cell proliferation and induced G0/G1 cell cycle arrest due to Sp1 transactivation. Regulation of nuclear Sp1 function by PARP-1 covers an important gap in our knowledge of mechanisms that control cell cycle.

## Results

### PARP-1 Promoted Cell Proliferation and Prevented Cell Cycle Arrest

**Figure 1:**
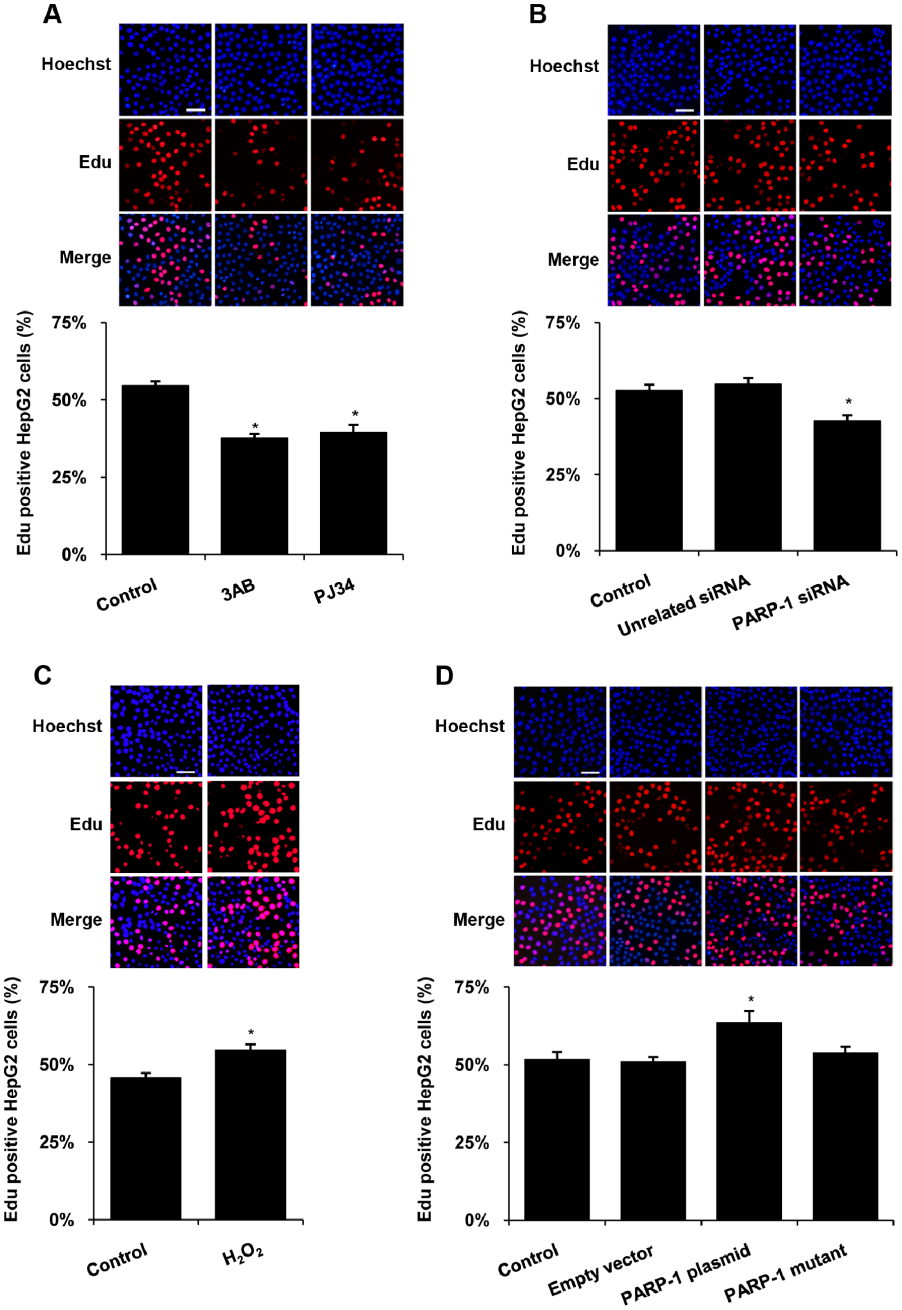
PARP-1 promoted proliferation of HepG2 cells. Cell proliferation assay was performed, in which Edu-labeled proliferative cells (red) and Hoechst-stained nuclei (blue) were observed under a fluorescent microscope (scale bar = 100 µm). Cells were treated with vehicles (PBS), 3AB (10 mmol/L, 24 h), PJ34 (10 µmol/L, 24 h) (A), PARP-1 siRNA (50 nmol/L, 48 h) (B), H_2_O_2_ (300 µmol/L, 0.5 h) (C), wild-type PARP-1 expressing plasmid (1 mg/L, 48 h) or PARP-1 mutant plasmid (1 mg/L, 48 h) (D) respectively. Data shown are representative of six independent experiments and are expressed as the mean±SEM, *p<0.05 compared to control group.

To explore the influence of PARP-1 on proliferation of liver cells, HepG2 cells were treated with PARP inhibitors 3AB (10 mmol/L) and PJ34 (10 µmol/L) for 24 hours. This concentration was chosen to avoid cell death. Cell proliferation assays showed that PARP inhibitor treatment dramatically impeded the proliferation of HepG2 cells (Figure 1A). Similar results were observed when PARP-1 was knocked down by PARP-1 siRNA (Figure 1B, Figure S1A). We then concluded that PARP-1 played a crucial role in the cell proliferation of hepatoma cells. To confirm our conjecture, the cultured HepG2 and Huh7 cells were exposed to H_2_O_2_, the enzymatic PARP-1 activator. As expected, H_2_O_2_ treatment promoted cell proliferation (Figure 1C). These results indicated that PARP-1 catalytic activity was involved in proliferation regulation. Furthermore, we built the full-length wild-type PARP-1 expression vector and mut-PARP-1 plasmid expressing an enzymatically inactive PARP-1 protein in which lysine 893 was substituted with isoleucine (K893I). Results showed that forced expression of wild-type PARP-1 dramatically promoted the proliferation of human hepatoma cells in a time-dependent manner, while the mut-PARP-1 failed to affect it (Figure 1D, Figure S1B). We obtained similar results in another hepatocarcinoma cell line Huh7 cells (Figure S2).

**Figure 2:**
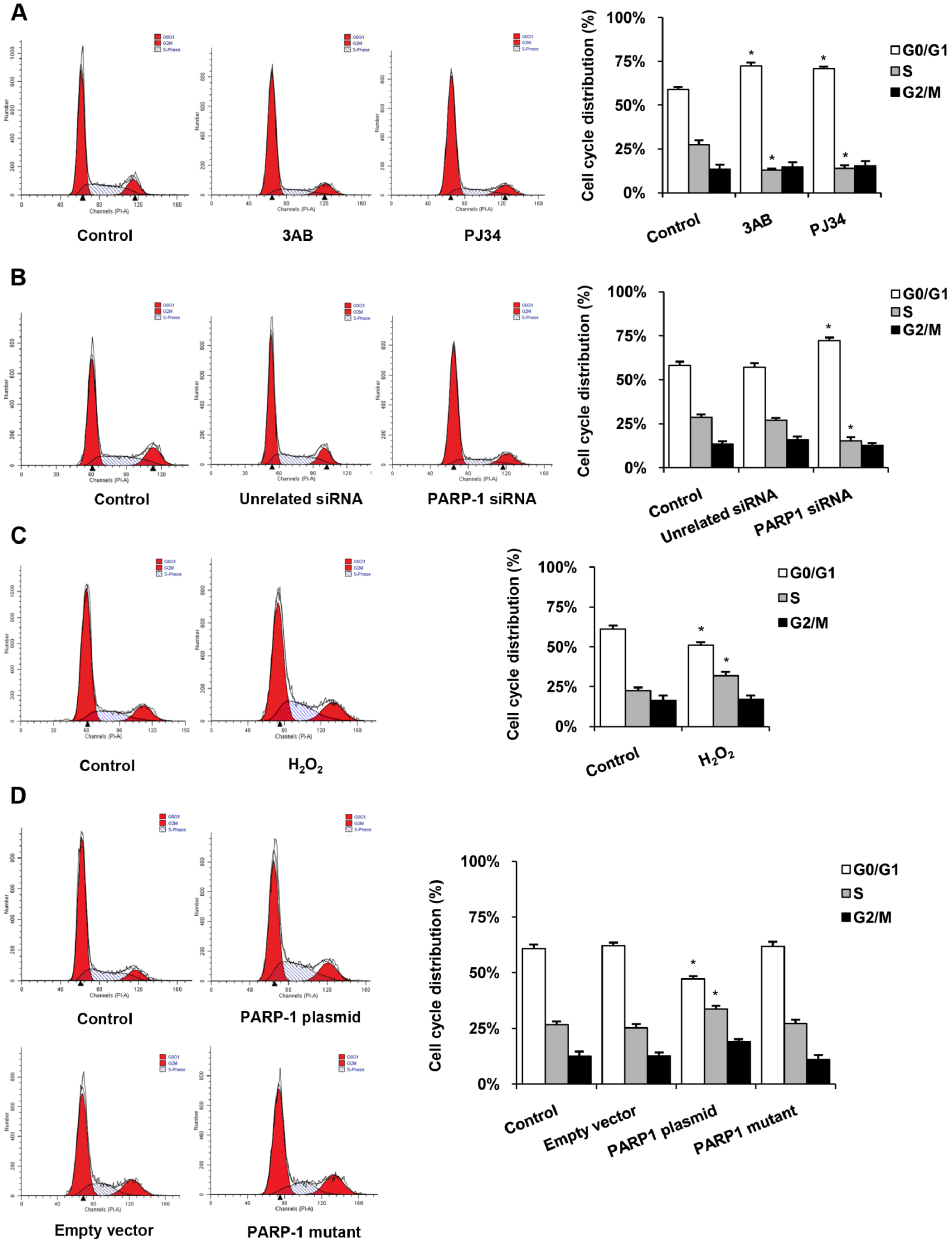
PARP-1 prevented cell cycle arrest. Cell cycle distribution was obtained by use of PI staining and analysis under a FACS Calibur flow cytometer. Cells were treated with vehicles (PBS), 3AB (10 mmol/L, 24 h), PJ34 (10 µmol/L, 24 h) (A), PARP-1 siRNA (50 nmol/L, 48 h) (B), H_2_O_2_ (300 µmol/L, 0.5 h) (C), PARP-1 expressing plasmid (1 mg/L, 48 h) or PARP-1 mutant plasmid (1 mg/L, 48 h) (D) respectively. Data shown are representative of six independent experiments and are expressed as the mean±SEM, *p<0.05 compared to control group.

Cell proliferation is controlled by the proliferative signaling pathways that induce quiescent cells to enter the proliferative cycle. In order to further characterize the role of PARP-1 on cell proliferation, HepG2 cells were prepared for cell cycle analysis. Administration of PARP inhibitor 3AB or PJ34 resulted in shorter S phase and higher proportion of cells in the G0/G1 phase (Figure 2A). PARP-1 depletion by PARP-1 siRNA also blocked entry of cells into the S phase (Figure 2B). On the contrary, H_2_O_2_ treatment resulted in a higher proportion of S phase (Figure 2C). Furthermore, overexpression of wild-type PARP-1, not mut-PARP-1 protein, promoted cell cycle process in G1-S checkpoint in HepG2 cells (Figure 2D). These results demonstrated that activated PARP-1 promoted proliferation of human hepatoma cells and prevented the cell cycle arrest at G0/G1 phase.

### PARP-1 Prevented Sp1-mediated Gene Transcription in HepG2 Cells

**Figure 3:**
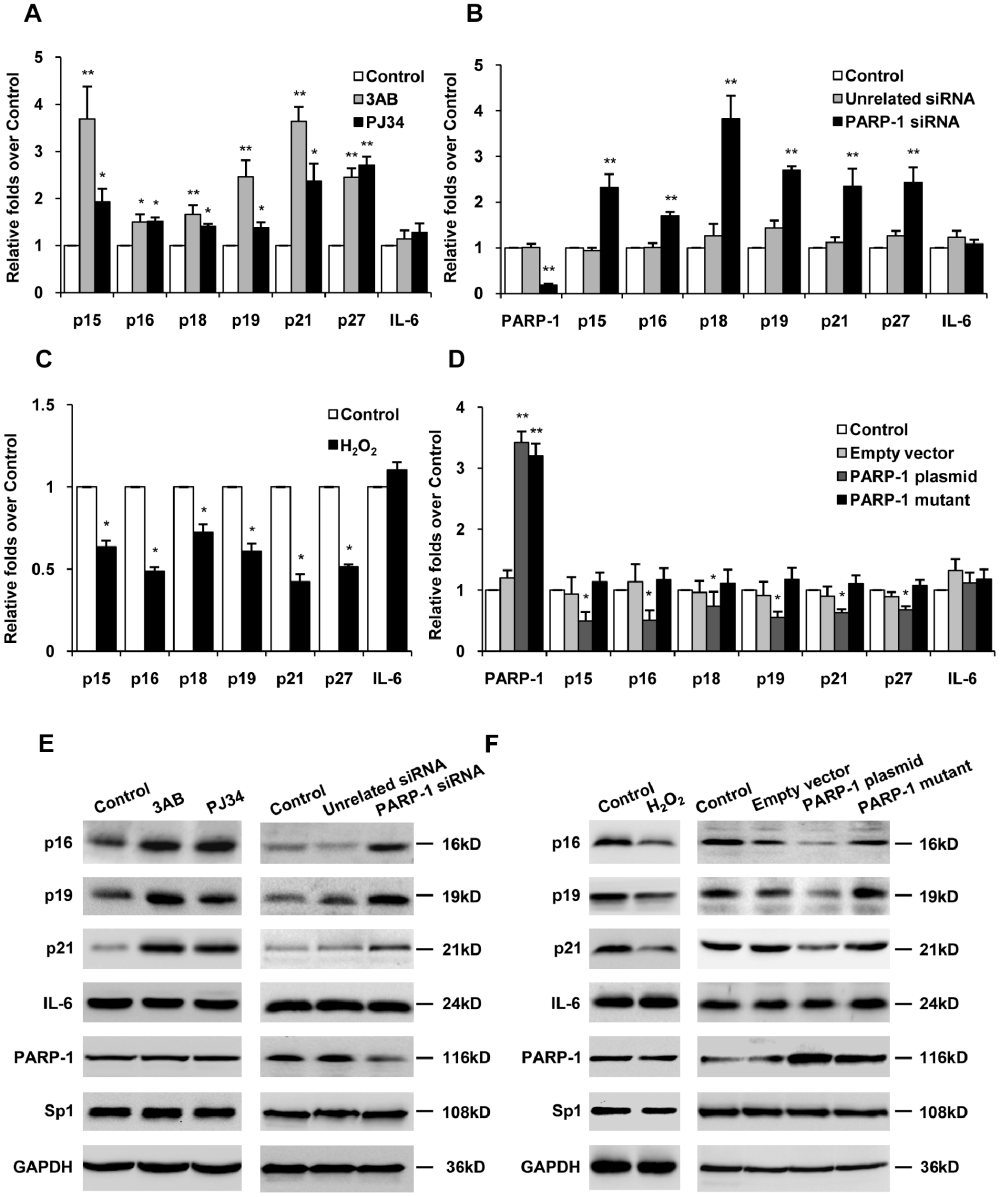
PARP-1 prevented Sp1-mediated gene transcription. Real-time PCR (A–D) and Western blotting (E–F) were used to detect expression of genes, as indicated in the figure. Cells were treated with vehicles (PBS), 3AB (10 mmol/L, 24 h), PJ34 (10 µmol/L, 24 h) (A, E), PARP-1 siRNA (50 nmol/L, 48 h) (B, E), H_2_O_2_ (300 µmol/L, 0.5 h) (C, F), PARP-1 expressing plasmid (1 mg/L, 48 h) or PARP-1 mutant plasmid (1 mg/L, 48 h) (D, F) respectively. Data shown are representative of six independent experiments and are expressed as the mean±SEM, *p<0.05, **p<0.01 compared to control group.

It has been demonstrated that Sp1 plays an important role in cell cycle progression by regulating its target genes [26]. We then investigated the role of PARP-1 in the expression of Sp1 target genes, such as p15, p16, p18, p19, p21 and p27 in HepG2 cells. Real time PCR showed that administration of PARP inhibitor, 3AB or PJ34 significantly increased mRNA expression of above mentioned genes (Figure 3A). Similar transcriptional enhancement was observed when PARP-1 was knockdown by siRNA (Figure 3B). Moreover, H_2_O_2_ treatment significantly inhibited the expression of these genes (Figure 3C).

To corroborate our listing data, HepG2 cells were transfected with plasmid expressing wild-type PARP-1 or mut-PARP-1 protein. In consistence with our speculation, overexpression of wild-type PARP-1 attenuated mRNA expression of Sp1 target genes, while the mut-PARP-1 failed to affect Sp1 mediated gene transcription (Figure 3D). Western blotting with p16, p19 or p21 antibody also showed similar results (Figure 3E, 3F).

### Inhibition of PARP-1 Decreased the Poly(ADP-ribosyl)ation of Sp1

**Figure 4:**
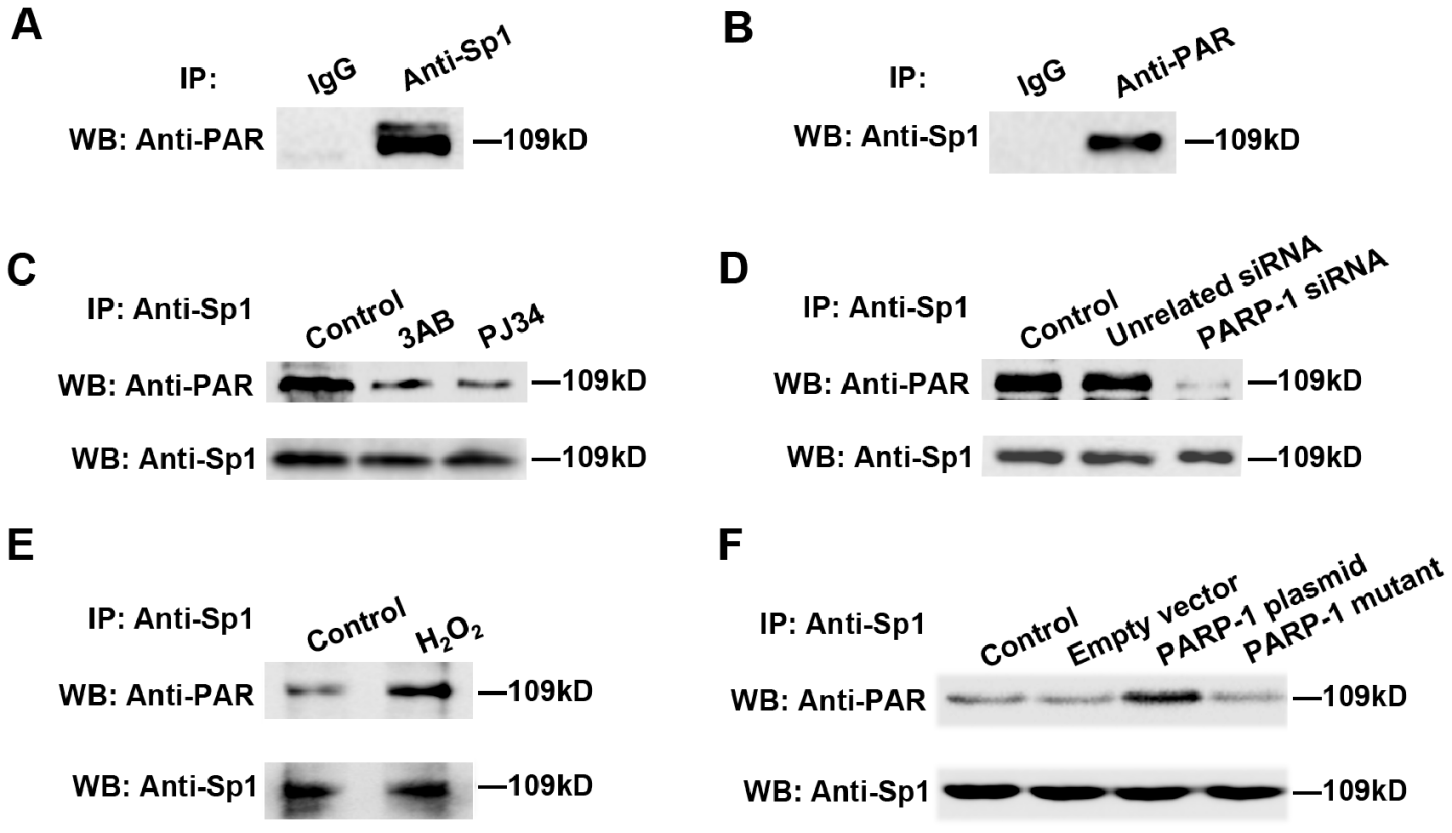
Inhibition of PARP-1 decreased the poly(ADP-ribosyl)ation of Sp1. (A) Immunoprecipitation of Sp1 bound proteins from HepG2 nuclear extracts, followed by western blotting using anti-PAR antibody. (B) Immunoprecipitation of poly(ADP-ribosyl)ated bound proteins from HepG2 nuclear extracts, followed by western blotting using anti-Sp1 antibody. Unspecific IgG served as negative control. (C–F) Immunoprecipitation of Sp1 bound proteins from HepG2 cells followed by western blotting using anti-PAR antibody. Cells were treated with vehicles (PBS), 3AB (10 mmol/L, 24 h), PJ34 (10 µmol/L, 24 h) (C), PARP-1 siRNA (50 nmol/L, 48 h) (D), H_2_O_2_ (300 µmol/L, 0.5 h) (E), PARP-1 expressing plasmid or PARP-1 mutant plasmid (1 mg/L, 48 h) (F) respectively. Sp1 served as loading control.

PARP-1 is known to be a poly(ADP-ribosyl)ating enzyme [27]. Transcriptional regulation by PARP-1 mainly involves in ADP-ribosylation-dependent mechanisms via physical interaction. Previous studies demonstrated that PARP-1 could bind to Sp1 directly [28], we then sought to determine whether Sp1 is poly(ADP-ribosyl)ated by PARP-1. Endogenous Sp1 of HepG2 cells was immunoprecipitated by anti-Sp1 antibody followed by western blotting using anti-PAR antibody. Results showed that Sp1 was poly(ADP-ribosyl)ated in HepG2 cells (Figure 4A). Similar results were obtained from the immunoprecipitation (IP) assays using anti-PAR antibody followed by western blotting with anti-Sp1 antibody (Figure 4B). Thereafter, the influences of 3AB or PJ34 on Sp1 poly(ADP-ribosyl)ation were investigated. IP assay with anti-Sp1 antibody followed by western blotting using anti-PAR antibody revealed that PARP inhibitors dramatically decreased the amount of poly(ADP-ribosyl)ated Sp1 (Figure 4C). To further detect whether or not Sp1 was poly(ADP-ribosyl)ated by PARP-1, HepG2 cells were treated with PARP-1 siRNA. Results showed that poly(ADP-ribosyl)ated Sp1 dramatically diminished in PARP-1 depletion cells (Figure 4D). Moreover, H_2_O_2_ treatment, as well as overexpressed wild-type PARP-1 protein, but not the mut-PARP-1 protein, promoted the poly(ADP-ribosyl)ation of Sp1 (Figure 4E, 4F).

To further explore the influence of PARP-1 on Sp1 expression, western blot assays with Sp1 antibody were performed. Results showed that the expression of Sp1 was unchanged when HepG2 cells were treated with PARP-1 inhibitors, H_2_O_2_, PARP-1 siRNA or PARP-1 plasmid (Figure 3E, 3F). These results indicated that the influence of PARP-1 inhibitors on the transactivation of Sp1 might be mediated by inhibiting its poly(ADP-ribosyl)ation level, not gene expression.

### Poly(ADP-ribosyl)ation Inhibited Sp1-DNA Complex Formation

**Figure 5:**
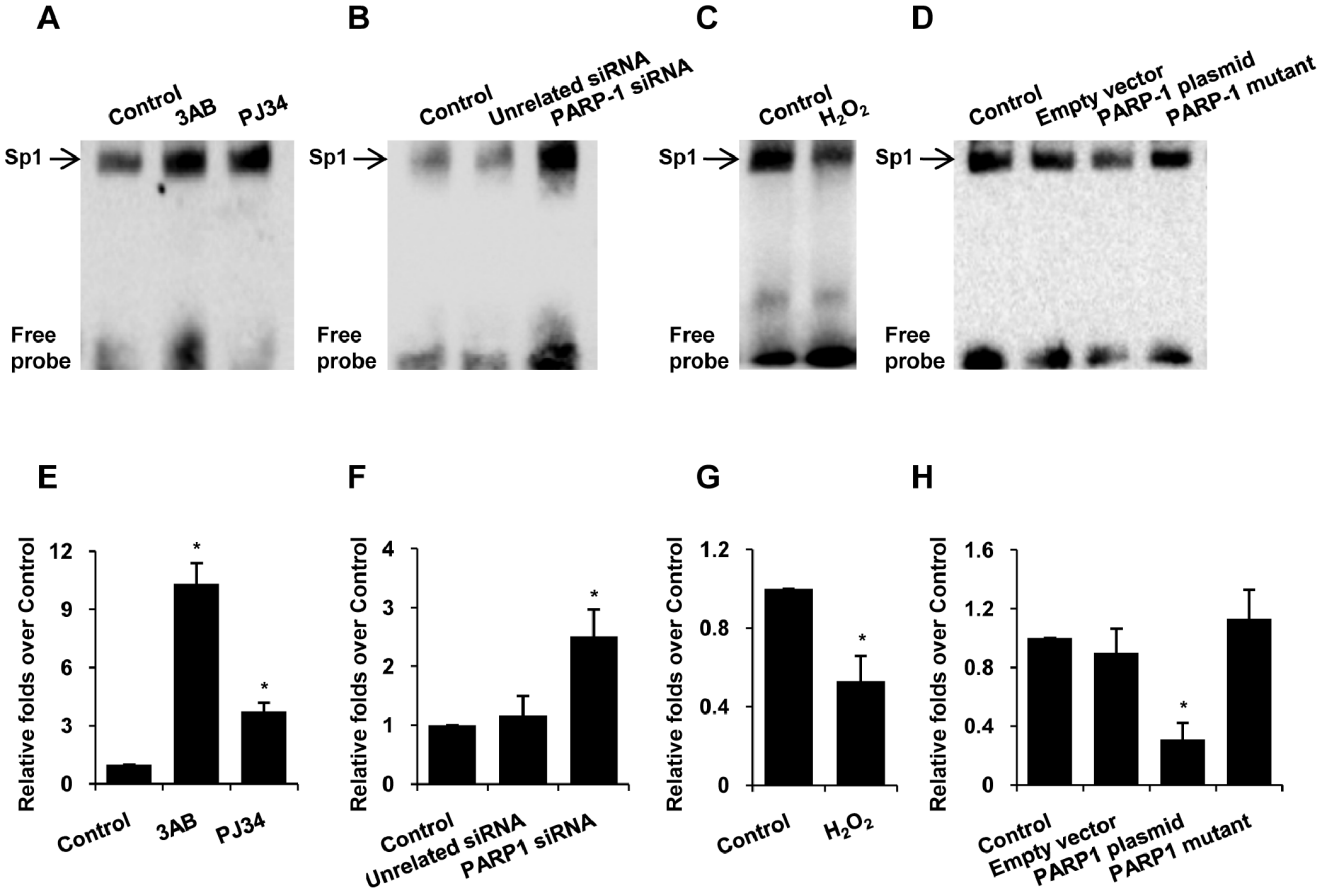
Poly(ADP-ribosyl)ation inhibited Sp1-DNA complex formation. (A–D) EMSA assay of HepG2 nuclear extracts was performed by use of an oligonucleotide probe containing Sp1 binding site. Cells were treated with vehicles (PBS), 3AB (10 mmol/L, 24 h), PJ34 (10 µmol/L, 24 h) (A), PARP-1 siRNA (50 nmol/L, 48 h) (B), H_2_O_2_ (300 µmol/L, 0.5 h) (C), PARP-1 expressing plasmid or PARP-1 mutant plasmid (1 mg/L, 48 h) (D) respectively. (E–H) ChIP assay was performed to see the recruitment of Sp1 to p21 promoter using specific anti-Sp1 antibody versus anti-IgG antibody. Quantitative real-time PCR was used to quantify the enrichment of endogenous p21 promoter loci. Cells were treated with vehicles (PBS), 3AB (10 mmol/L, 24 h), PJ34 (10 µmol/L, 24 h) (E), PARP-1 siRNA (50 nmol/L, 48 h) (F), H_2_O_2_ (300 µmol/L, 0.5 h) (G), PARP-1 expressing plasmid or PARP-1 mutant plasmid (1 mg/L, 48 h) (H) respectively. Data shown are representative of six independent experiments and are expressed as the mean±SEM, *p<0.05 compared to control group.

Sp1 regulates gene expression through binding to specific sites in the promoter of its target genes. To investigate the influence of poly(ADP-ribosyl)ation on the DNA binding activity of Sp1, electrophoretic mobility shift assay (EMSA) was performed by use of an oligonucleotide probe containing Sp1 binding site (Sp1 response elements). Results showed that inhibition of PARP-1 by 3AB, PJ34 or PARP1 siRNA was of benefit for the formation of Sp1-DNA complex (Figure 5A, 5B). Conversely, H_2_O_2_ treatment inhibited Sp1 binding to DNA (Figure 5C). Forced increase of poly(ADP-ribosyl)ation by wild-type PARP-1 transfection led to decreased Sp1-DNA complex formation, while mut-PARP-1 transfection had no such effect (Figure 5D). In line with our results, *in vitro* poly(ADP-ribosyl)ation assay also demonstrated that incubation of nuclear extracts from non-treated cells with NAD^+^ and active DNA inhibited the formation of Sp1-DNA complex [29].

Moreover, chromatin immunoprecipitation (ChIP) experiments using specific anti-Sp1 antibody demonstrated that inhibition of PARP-1 activity or depletion of PARP-1 by siRNA assisted the recruitment of Sp1 to p21 promoter in HepG2 cells (Figure 5E, 5F). Administration of H_2_O_2_ or transfection of wild-type PARP-1 plasmid decreased the enrichment of the p21 promoter fragments to Sp1 (Figure 5G, 5H). These results illustrated that poly(ADP-ribosyl)ation of Sp1 attenuated Sp1 binding to DNA in its target promoters.

### PARP-1 Prevented Sp1-mediated Transcriptional Activation

**Figure 6:**
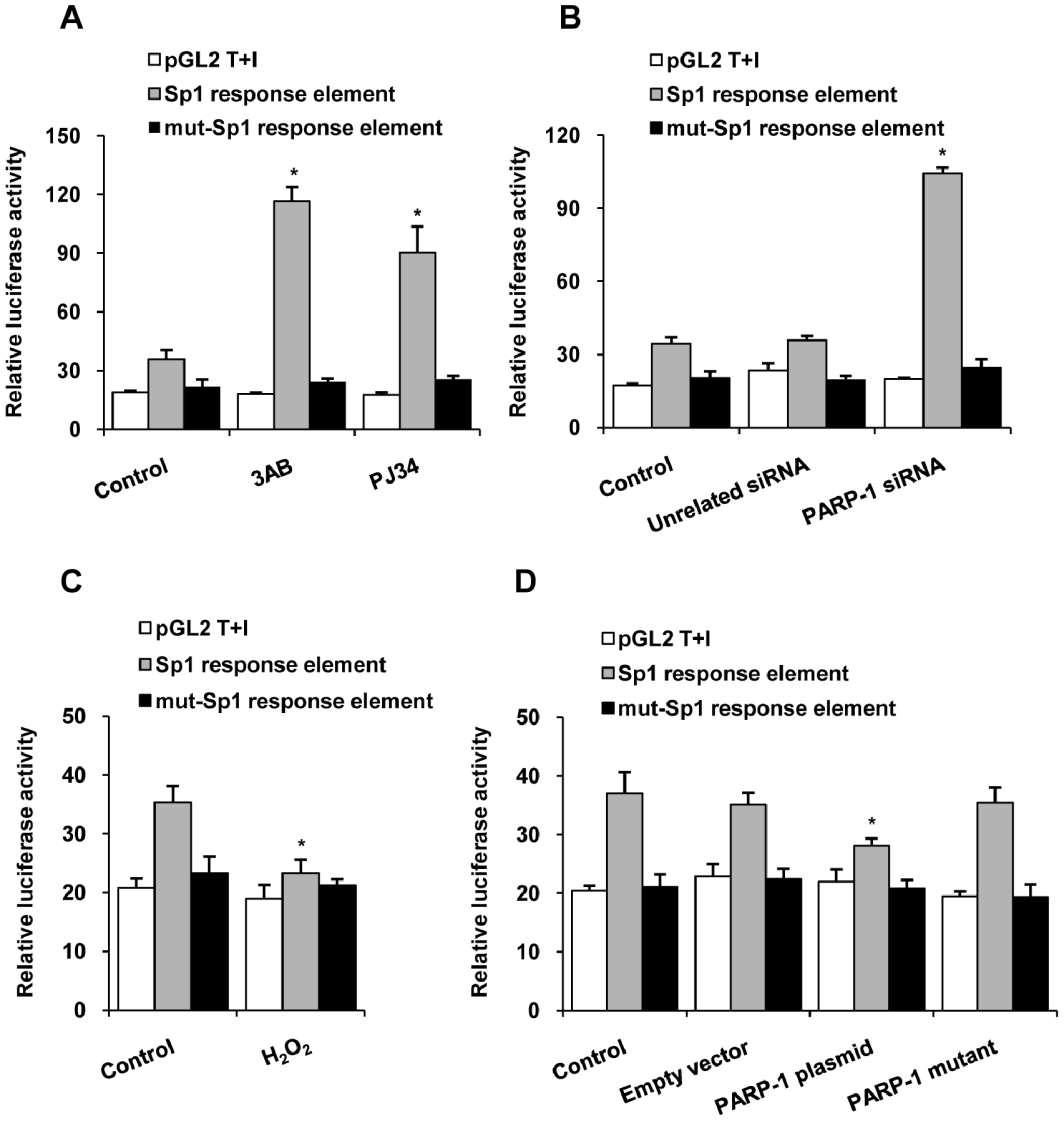
Inhibition of PARP-1 prevented Sp1-mediated transactivation. Luciferase assay was used to detect wild type or mutant Sp1-responsive luciferase reporter activity in HepG2 cell. The empty vector pGL2T+I served as negative control. Cells were treated with vehicles (PBS), 3AB (10 mmol/L, 24 h), PJ34 (10 µmol/L, 24 h) (A), PARP-1 siRNA (50 nmol/L, 48 h) (B), H_2_O_2_ (300 µmol/L, 0.5 h) (C), PARP-1 expressing plasmid or PARP-1 mutant plasmid (1 mg/L, 48 h) (D) respectively. Data representative of six independent experiments and are expressed as the mean±SEM, *p<0.05 compared to control group.

In the nucleus, Sp1 directly binds to Sp1 response elements at −71 to −86 of the p21 promoter to regulate p21 transcription [30]. In a simplified transcription-based situation, such as the luciferase assay, the model is straightforward. A wild type luciferase reporter which contains four times of truncated p21 promoter (base −71 to −86) and mut-Sp1-response reporter plasmid were constructed. Results showed that inhibition of PARP activity by 3AB or PJ34, or knockdown of PARP-1 by siRNA increased the wild type luciferase reporter activity in HepG2 cells under basic conditions, while the Sp1 depletion could abrogate these effects (Figure 6A, 6B, Figure S3, Figure S4). Conversely, H_2_O_2_ or forced expression of wild-type PARP-1 reduced the luciferase activity in HepG2 cells, while the mut-PARP-1 failed to affect the Sp1 transcription (Figure 6C, 6D). Moreover, both H_2_O_2_ treatment and over-expression of wild-type PARP-1 protein did not affect this luciferase activity in Sp1 knock-down HepG2 cells (Figure S3). In addition, luciferase activity of mut-Sp1-response reporter plasmid did not change basically when HepG2 cells were treated with PARP-1 inhibitors, PARP-1 siRNA, H_2_O_2_ or PARP-1 plasmid (Figure 6). All these results illustrated that inhibition of PARP-1 promoted Sp1 transcriptional activation.

## Discussion

Sp1 is G1 cell cycle phase specific transcription factor. It accumulates in most types of cancer cells and works as an essential modulator in regulating cell growth, angiogenesis, and survival in various cancers [11], [12], [13], [14], [15], [16], [17]. In this study, we presented the first evidence that inhibition of PARP-1 induced cell cycle arrest at the G1-S checkpoint in hepatoma cells. These protective effects are mediated via activation of Sp1 signaling pathway.

PARP-1 plays a critical role in cell proliferation by exerting distinct functions in different cell cycle phases. Simbulan-Rosenthal CM *et al* has demonstrated that PARP-1 promotes quiescent cells to re-entry the cell cycle as the cofactor of E2F-1 [24]. Another PARP-1 inhibitor olaparib has potent antitumor activity in breast cancer cells [31]. Consistent with these researches, we found that inhibition of PARP-1 by PARP inhibitors or PARP-1 siRNA significantly increased the expression of Sp1 target genes such as p21 and p27, resulting in G0/G1 cell cycle arrest and decreased proliferative ability of HepG2 cells. However, the mut-PARP-1 failed to affect the proliferation of HepG2 cells. We then speculated that the regulatory functions of PARP-1 in the proliferation of hepatoma cells might base on the enzymatic activity of PARP-1.

PARP-1 is the predominant enzyme of PARP family, responsible for about 90% of cellular PAR formation. As with many other nuclear proteins, Sp1 serves as integrating platforms for a variety of stimuli and is the target for post-translational modifications, such as glycosylation, ubiquitination, sumoylation, acetylation and phosphorylation [32], [33], [34], [35], [36]. Previous results also showed that *in vitro* incubation of nuclear extracts with NAD^+^ decreased the DNA binding activity of Sp1 [28], [29]. In addition, we have shown here that Sp1 was poly(ADP-ribosyl)ated in HepG2 cells. All these results identified poly(ADP-ribosyl)ation as another important post-transcriptional modification of Sp1. The PARP-1 depletion cells were previously confirmed to be devoid of PARP-1 and PAR. Although other members of the PARP family have been found, their activity still could not fully compensate for PARP-1 depletion [37]. Accordingly, similar to PARP inhibitors, PARP-1 siRNA also abolished the poly(ADP-ribosyl)ation of Sp1.

Poly(ADP-ribosyl)ation forms definitive structures on other molecules through intramolecular interactions. These structures have the potential for noncovalent attractive (or repulsive) interactions with these receptors [27]. Increasing researches demonstrate that the activity of various transcription factors, such as NF-κB, CREB and PPARγ, was severely reduced in the presence of NAD^+^ [38], [39]. Sp1 was poly(ADP-ribosyl)ated by wild type PARP-1 *in vivo*, indicating that PARP-1 exerted a direct effect on Sp1 transcriptional activation. In this case, both PARP-1 inhibitors and PARP-1 siRNA abolished poly(ADP-ribosyl)ation of Sp1. We then conjectured that the state of poly(ADP-ribosyl)ation determined the DNA binding activity of Sp1 itself. To confirm our conjecture, inhibition of PARP-1 activity by PARP inhibitors or PARP-1 siRNA increased DNA binding activity of Sp1. All these data suggest that PARP-1 attenuates the DNA affinity of Sp1 through poly(ADP-ribosyl)ation of Sp1.

It has been shown that cell cycle inhibitor p21 is the direct target gene of Sp1. Transactivation of p21 requires the presence of Sp1 response elements. Our analysis of the p21 promoter revealed that PARP-1 induced poly(ADP-ribosyl)ation of Sp1 and blocked p21 transactivation. As Sp1 response elements have been identified in promoter regions of several growth and cell cycle regulated genes, the regulatory effect of PARP-1 on p21 transactivation probably extends to these genes. Up to this point, we got enough reliable evidence that poly(ADP-ribosyl)ation of Sp1 not only lowered its positive regulatory influence on gene transcription but also its capacity to physically interact with its high affinity sites in target gene promoter.

In summary, our work demonstrated that PARP-1 inhibition blocked the growth of the human hepatoma cell line. Inhibition of PARP-1 caused G0/G1 cell cycle arrest by activating Sp1 pathway, and this regulatory function mainly depended on its state of post modification. The poly(ADP-ribosyl)ation-induced suppression of Sp1 by PARP-1 unraveled a novel function for PARP-1 in gene regulation. This finding also exerted a new mechanism how PARP-1 regulates cell proliferation and cell cycle checkpoint in hepatoma cells. Moreover, targeting PARP-1 may be a promising therapeutic approach against human hepatocellular carcinoma.

## Materials and Methods

### Cell Culture and Transfection

The human HepG2 cell line (ATCC HB 8065) and Huh7 cell line (JCRB 0403) were purchased from the Cell Bank of Type Culture Collection of Chinese Academy of Sciences. HepG2 cells were cultured in RPMI 1640 medium (Gibco) and Huh7 cells in Dulbecco's Modified Eagle medium (Gibco), supplemented with 10% (v/v) fetal bovine serum (Gibco) and 1% antibiotics (penicillin and streptomycin) under humidified conditions with 5% CO_2_ at 37°C. Seeded in 6-well plates at 70% confluence, cells were then treated with the following agents: 10 mmol/L 3AB (Sigma, 24 h, IC_50_∶33 µmol/L [40]), 10 µmol/L PJ34 (Alexis Biochemicals, 24 h, IC_50_∶1.0 µmol/L [41]), 300 µmol/L H_2_O_2_ (Sigma, 0.5 h) or vehicle control (PBS).

### Plasmid Construction, RNA Interference and Transfection

The Full-length wild type cDNA of human PARP-1 was cloned by RT-PCR from HepG2 cells. The PARP-1 expression vector (PARP-1 plasmid) was constructed in the mammalian expression vector p3flag-CMV (Sigma). A catalytically inactive mutant of PARP-1 (PARP-1 mutant) in which lysine893 is substituted by isoleucine (K893I) was generated as previously described by using the QuickChange site directed mutagenesis kit (Stratagene) [42].

Small interfering RNAs (siRNAs) for PARP-1 (sense 5′-GGA UGA UCU UCG ACG UGG A-3′, antisense 5′-UCC ACG UCG AAG AUC AUC C-3′) and for Sp1 (sense 5′-CCA ACA GAU UAU CAC AAA U-3′, antisense 5′-AUU UGU GAU AAU CUG UUG G -3′) were synthesized by RiBoBio Co. Ltd.

Cells were seeded at 70% confluence for siRNA (50 nmol/L) or plasmid (1 mg/L) transfection separately, using Lipofectamine 2000 (Invitrogen) according to the manufacturer’s protocol. The efficiency of PARP-1 siRNA or PARP-1 plasmid was detected by real-time RT-PCR assay as well as western blotting shown in Figure 3.

### Cell Proliferation Assay

Cell proliferation was determined by use of Cell-Light EdU DNA Cell Proliferation Kit (RiBoBio Co. Ltd) [43]. Briefly, cells (1×10^4^∼1×10^5^) were cultured in 24-well plates. After stimulation, cells were exposed to 50 µmol/L EdU for 2 h at 37°C. Then cells were fixed in 4% formaldehyde for 30 min at room temperature and permeabilized in 0.5% Triton X-100 for 10 min. Each well was washed with PBS, followed by incubation with 200 µl 1×Apollo reaction cocktail for 30 min. DNA was then stained with 1×Hoechst 33342 (200 ml per well) for 30 min and imaged under a fluorescent microscope (Olympus Optical Co. LTD). Cell proliferation was quantified with EdU incorporation counting a minimum of 10 fields with 100 nuclei per condition.

### Propidium Iodide (PI)/FACS Analysis

Cells were seeded in 6-well plates and grew for 24 h. After stimulation, cells were collected and fixed in ice-cold 70% ethanol overnight. Then, cells were incubated in PBS containing 250 µg/mL RNase for 30 min at 37°C and incubated in PBS containing 50 µg/mL PI (Sigma) for 30 min in the dark. Cell cycle distribution was analyzed using a FACS Calibur flow cytometer (BD Biosciences).

### Quantitative Real-time PCR Analysis

RNA was extracted from cultured cells using Trizol reagent (Takara) according to the manufacturer's instruction. RNA was then reverse transcribed using RNA PCR Kit (Takara). Quantitative RT-PCR was performed on ABI PRISM 7900 Sequence Detector system (Applied Biosystem) using SYBR Green I Assay (Takara). Relative gene expression level (the amount of target, normalized to endogenous control gene) was calculated using the comparative Ct method formula 2^−ΔΔCt^. GAPDH was used as endogenous control. The primers used in this study were listed in Table 1.

**Table 1:** The sequences of primers for quantitative real time RT-PCR used in this study.

Name	Forward primer sequence (5′–3′)	Reversed primer sequence (5′–3′)
GAPDH	GGCCTCCAAGGAGTAAGACC	CTGTGAGGAGGGGAGATTCA
PARP-1	AAGGCGAATGCCAGCGTTAC	GCACTCTTGGAGACCATGTCA
p15	GCGGGGACTAGTGGAGGA	CATCATCATGACCTGGATCG
p16	GCTGCCCAACGCACCGA	CATTCCTCTTCCTTGGCTTCCC
p18	ATTGCCAGGAGACTGCTAC	CCCTTATGGTTCCGATGC
p19	GACCCAAGGGCAGAGCAT	TCTTATTGATTTGGGACGCT
p21	ACCGAGACACCACTGGAGGG	CGAGGCACAAGGGTACAAGACA
p27	AACGTGCGAGTGTCTAACGG	CCTCTAGGGGTTTGTGATTCT
IL-6	GAAGAGCGCCGCTGAGAAT	GTGCAGAGGGTTTAATGTCAACT

### Preparation of Whole Extracts and Nuclear Extracts

Whole extracts and nuclear extracts were prepared as described previously [39]. Protein extracts were quantified using the Bradford assay.

### Western Blotting

Protein extracts were loaded onto 9% sodium dodecyl sulfate-polyacrylamide gel electrophoresis (SDS-PAGE) gels and then transferred onto nitrocellulose membranes. Membranes were blocked in 5% non-fat milk diluted in Tris-buffered saline with Tween 20 (TBST) for 3 h at room temperature, followed by primary antibody incubations overnight at 4°C. Antibodies used were anti-Sp1 (1∶1000, Santa Cruz), anti-p16 (1∶1000, Abcam), anti-p19 (1∶1000, Abcam), anti-p21 (1∶1000, Abcam), anti-IL-6 (1∶500, Santa Cruz), anti-PARP-1 (1∶1000, R&D), anti-PAR (1∶1000, Trevigen) and anti-GAPDH (1∶500, Santa Cruz). Then membranes were washed 3×15 min with TBST and incubated with peroxidase-conjugated secondary antibody (1∶3000, Cell Signaling Technology) at room temperature for 2 h. The membrane was washed 3×15 min with TBST again and specific band was detected with chemiluminescence assay using ECL detection reagents (Pierce).

### Immunoprecipitation (IP) Assay

IP assay was performed as described previously [44]. Briefly, 500 µg of nuclear extracts were incubated with the indicated antibodies (anti-Sp1, anti-PAR, or unspecific IgG respectively) at 4°C for 1 h, and protein-G agarose at 4°C for 12 h. The immunoprecipitates were pelleted by centrifugation at 5000 g for 1 minute and washed 4 times with lysis buffer, followed by SDS-PAGE analysis. Unspecific IgG was used as negative control.

### Electrophoretic Mobility Shift Assay (EMSA)

DNA-protein interaction was detected using LightShiftTM Chemiluminescent EMSA kit (Pierce) according to the manufacturer's protocol. The sequence of the oligonucleotide probe containing Sp1 binding site was: 5′-ATT CGA TCG GGG CGG GGC GAG C-3′. Biotin was labeled at the 5′ end of the oligonucleotides. After incubation of nuclear extracts with Sp1 oligonucleotide probe at room temperature for 20 minutes, reaction mixture was subjected to 6% native polyacrylamide gel electrophoresis and thereafter transferred to nylon membranes (Pierce) which were immediately cross-linked on a UV transilluminator. Bands were then detected with chemiluminescent method following manufacturer’s protocol.

### Chromatin Immunoprecipitation (ChIP) Assay

ChIP experiments were performed as previously described [45]. In ChIP experiments, HepG2 cells were sonicated and the lysates were immunoprecipitated using 5 µg Sp1 antibody (Santa Cruz) or IgG (Santa Cruz, as negative control). The sheared DNA was extracted with a DNA extraction kit for further quantitative real-time PCR analysis. The chromosomal DNA input and ChIP DNA with nonspecific IgG were subjected to the same PCR amplification. The primer sequences spanning the Sp1 binding site in the endogenous p21 promoter were AGT GCC AAC TCA TTC TCC AAG (sense) and GAC ACA TTT CCC CAC GAA GT (anti-sense) [46].

### Luciferase Assay

The Sp1-responsive luciferase reporter plasmid and its empty vector pGL2T+I were kindly provided by Dr. Moshe Szyf (department of pharmacology and therapeutics, McGill university, Canada), which contains four times of the consensus Sp1 binding site corresponding to the base −71 to −86 of the p21 promoter (sequence: GGT CCC GCC TCC TTG A) [30]. The mutant reporter plasmid (sequence: GGT CCC G**GA** TCC TTG A) was obtained by using the QuickChange site directed mutagenesis kit (Stratagene). The wild type or mutant Sp1-responsive luciferase reporter plasmid (0.5 µg) was cotransfected with pRL-SV40 plasmid (6 ng, internal control for normalization of transfection efficiency, Promega) into HepG2 cells using lipofectamine 2000 (Invitrogen). The empty vector pGL2T+I was used as control. The luciferase activity was determined with Dual Luciferase Reporter Assay Kit (Promega) according to the manufacturer’s instruction. All luciferase activity was normalized with the renilla luciferase activity.

### Statistic Analysis

All values were shown as mean±SEM of at least six independent experiments. Statistical significance was estimated by one-way ANOVA followed by Least-significant difference multiple comparison tests. *p* values <0.05 were considered statistically significant. All statistical analyses were performed with SPSS software (version 11.0, SPSS Inc).

## Supporting Information

Figure S1 **PARP-1 promoted proliferation of HepG2 cells.** Cell proliferation assay was performed, in which Edu-labeled proliferative cells (red) and Hoechst-stained nuclei (blue) were observed under a fluorescent microscope (scale bar = 100 µm). Cells were transfected with PARP-1 siRNA (50 nmol/L) (A), PARP-1 expressing plasmid (1 mg/L) or PARP-1 mutant plasmid (1 mg/L) (B) respectively, and were analyzed in different time points as indicated in the figure. Data shown are representative of six independent experiments and are expressed as the mean±SEM, *p<0.05 compared to control group.
(TIF)

Figure S2 **PARP-1 promoted proliferation of Huh7 cells.** Cell proliferation assay was performed, in which Edu-labeled proliferative cells (red) and Hoechst-stained nuclei (blue) were observed under a fluorescent microscope (scale bar = 100 µm). Cells were treated with vehicles (PBS), 3AB (10 mmol/L, 24 h), PJ34 (10 µmol/L, 24 h), H_2_O_2_ (300 µmol/L, 0.5 h) (A), PARP-1 siRNA (50 nmol/L, 12/24/48 h) (B), PARP-1 expressing plasmid (1 mg/L, 12/24/48 h) or PARP-1 mutant plasmid (1 mg/L, 12/24/48 h) (C) respectively. Data shown are representative of six independent experiments and are expressed as the mean±SEM, *p<0.05 compared to control group.
(TIF)

Figure S3 **Effect of PARP-1 inhibition on Sp1-mediated transactivation was abolished when Sp1 was knocked down.** Luciferase assay was used to detect wild-type Sp1-responsive luciferase reporter activity in HepG2 cell. The empty vector pGL2T+I served as negative control. Cells were transfected with Sp1 siRNA (50 nmol/L, 24 h) or unrelated siRNA (50 nmol/L, 24 h), followed by treatment of vehicles (PBS), 3AB (10 mmol/L, 24 h), PJ34 (10 µmol/L, 24 h) (A), PARP-1 siRNA (50 nmol/L, 48 h) (B), H_2_O_2_ (300 µmol/L, 0.5 h) (C), PARP-1 expressing plasmid or PARP-1 mutant plasmid (1 mg/L, 48 h) (D) respectively. Data shown are representative of six independent experiments and are expressed as the mean±SEM.
(TIF)

Figure S4 **PARP-1 inhibitors prevented Sp1-mediated transactivation in a dose-dependent manner.** Luciferase assay was used to detect wild-type Sp1-responsive luciferase reporter activity in HepG2 cell. The empty vector pGL2T+I served as negative control. Cells were treated with vehicles (PBS), 3AB (2.5/5/10 mmol/L, 24 h), or PJ34 (2.5/5/10 µmol/L, 24 h) respectively. Data shown are representative of six independent experiments and are expressed as the mean±SEM. *p<0.05 compared to control group.
(TIF)
