# Supplementary figures and images for: Identification of Poly(ADP-Ribose) Polymerase-1 as a Cell Cycle Regulator through Modulating Sp1 Mediated Transcription in Human Hepatoma Cells
Source: PLoS One. 2013 Dec 19;8(12):e82872. doi: 10.1371/journal.pone.0082872 (PMC3868549; doi:10.1371/journal.pone.0082872)

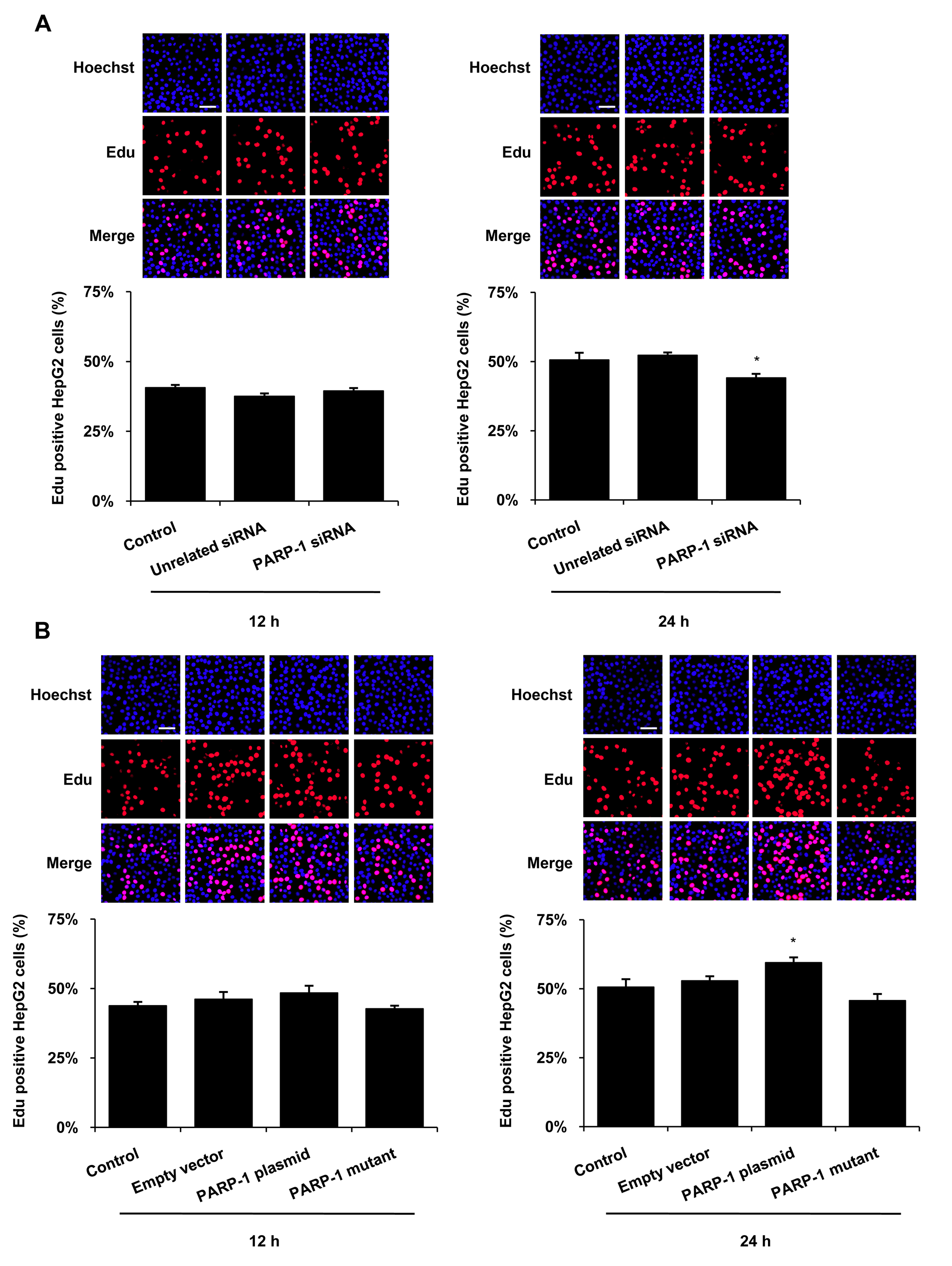

Supplement: Figure S1 — PARP-1 promoted proliferation of HepG2 cells. Cell proliferation assay was performed, in which Edu-labeled proliferative cells (red) and Hoechst-stained nuclei (blue) were observed under a fluorescent microscope (scale bar = 100 µm). Cells were transfected with PARP-1 siRNA (50 nmol/L) (A), PARP-1 expressing plasmid (1 mg/L) or PARP-1 mutant plasmid (1 mg/L) (B) respectively, and were analyzed in different time points as indicated in the figure. Data shown are representative of six independent experiments and are expressed as the mean±SEM, *p<0.05 compared to control group. (TIF) [file pone.0082872.s001.tif]

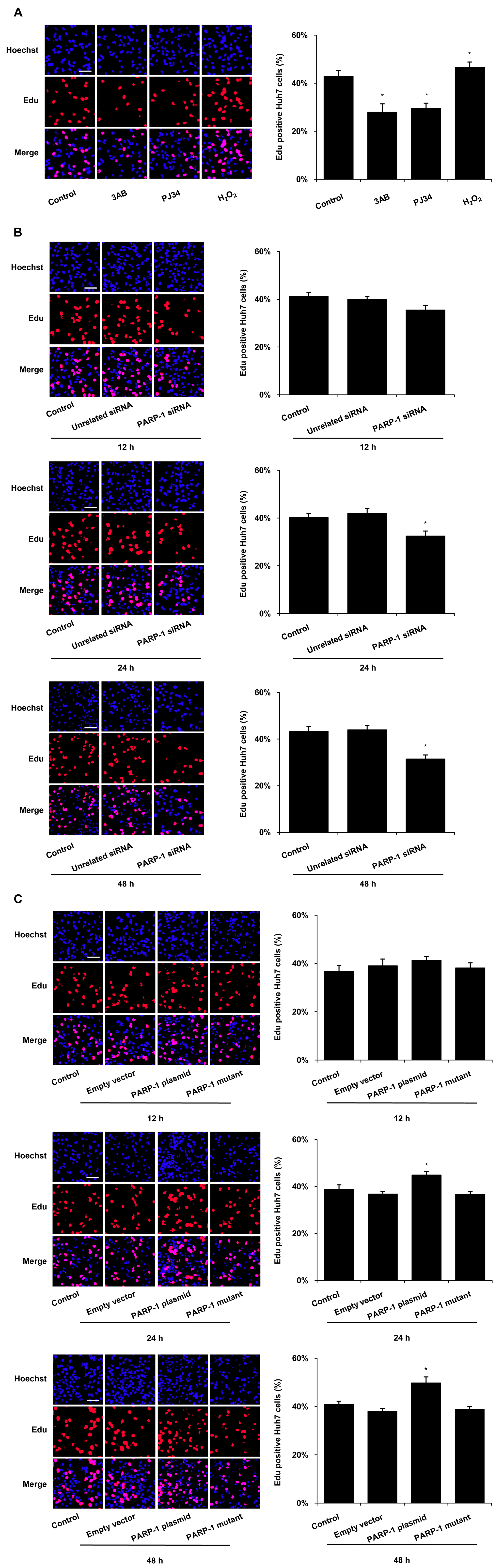

Supplement: Figure S2 — PARP-1 promoted proliferation of Huh7 cells. Cell proliferation assay was performed, in which Edu-labeled proliferative cells (red) and Hoechst-stained nuclei (blue) were observed under a fluorescent microscope (scale bar = 100 µm). Cells were treated with vehicles (PBS), 3AB (10 mmol/L, 24 h), PJ34 (10 µmol/L, 24 h), H2O2 (300 µmol/L, 0.5 h) (A), PARP-1 siRNA (50 nmol/L, 12/24/48 h) (B), PARP-1 expressing plasmid (1 mg/L, 12/24/48 h) or PARP-1 mutant plasmid (1 mg/L, 12/24/48 h) (C) respectively. Data shown are representative of six independent experiments and are expressed as the mean±SEM, *p<0.05 compared to control group. (TIF) [file pone.0082872.s002.tif]

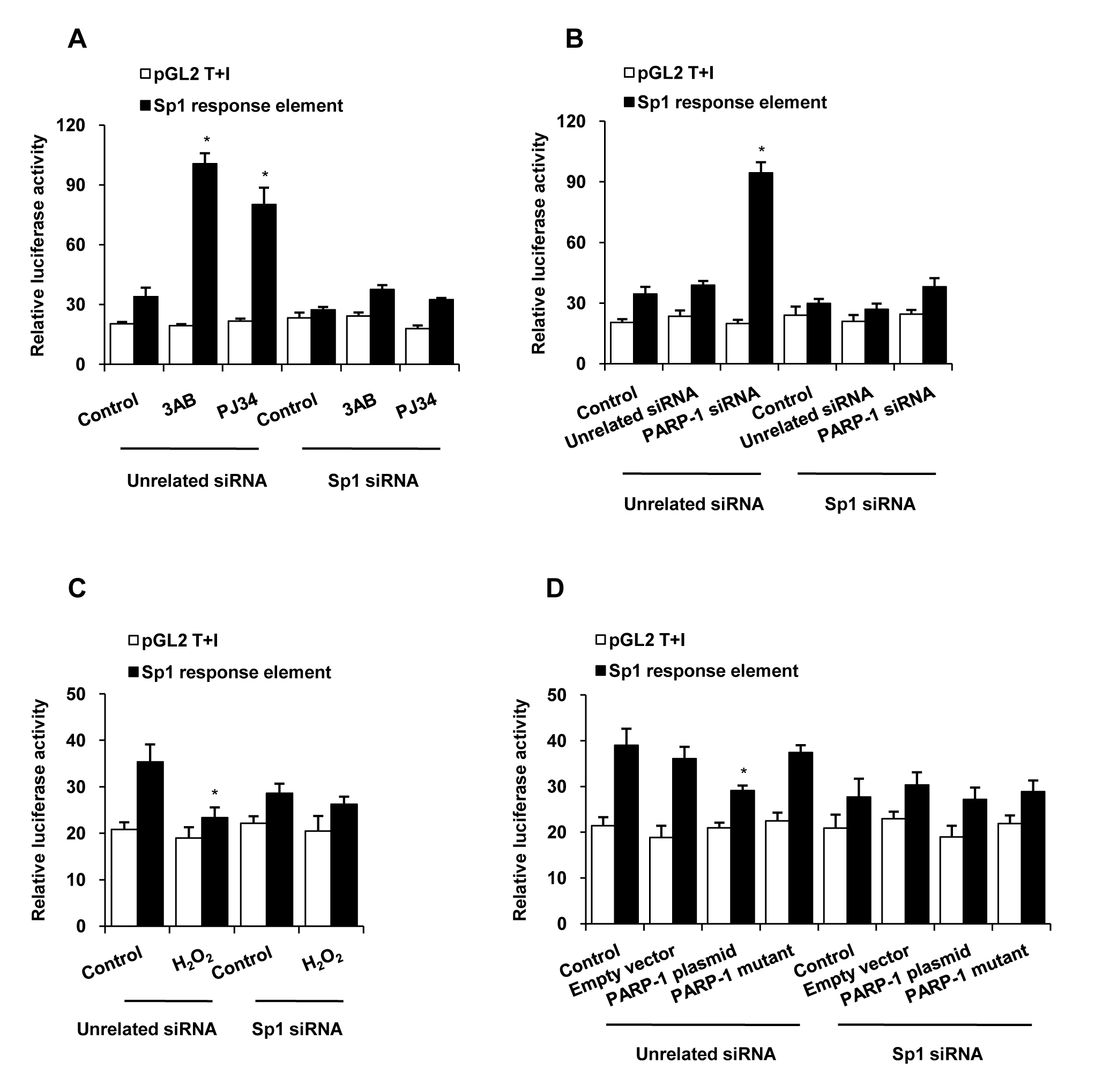

Supplement: Figure S3 — Effect of PARP-1 inhibition on Sp1-mediated transactivation was abolished when Sp1 was knocked down. Luciferase assay was used to detect wild-type Sp1-responsive luciferase reporter activity in HepG2 cell. The empty vector pGL2T+I served as negative control. Cells were transfected with Sp1 siRNA (50 nmol/L, 24 h) or unrelated siRNA (50 nmol/L, 24 h), followed by treatment of vehicles (PBS), 3AB (10 mmol/L, 24 h), PJ34 (10 µmol/L, 24 h) (A), PARP-1 siRNA (50 nmol/L, 48 h) (B), H2O2 (300 µmol/L, 0.5 h) (C), PARP-1 expressing plasmid or PARP-1 mutant plasmid (1 mg/L, 48 h) (D) respectively. Data shown are representative of six independent experiments and are expressed as the mean±SEM. (TIF) [file pone.0082872.s003.tif]

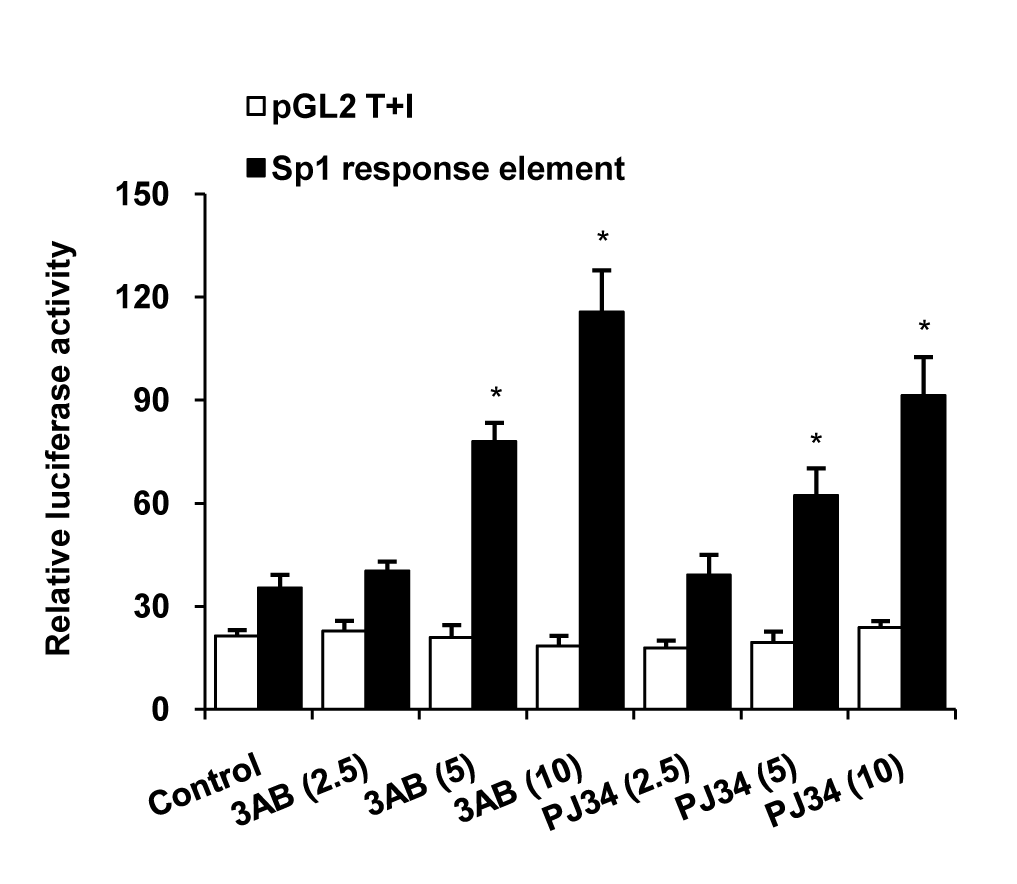

Supplement: Figure S4 — PARP-1 inhibitors prevented Sp1-mediated transactivation in a dose-dependent manner. Luciferase assay was used to detect wild-type Sp1-responsive luciferase reporter activity in HepG2 cell. The empty vector pGL2T+I served as negative control. Cells were treated with vehicles (PBS), 3AB (2.5/5/10 mmol/L, 24 h), or PJ34 (2.5/5/10 µmol/L, 24 h) respectively. Data shown are representative of six independent experiments and are expressed as the mean±SEM. *p<0.05 compared to control group. (TIF) [file pone.0082872.s004.tif]
